# Impact of Rectangular Collimation on Quality of Intraoral Radiographs: Findings from a Clinical Audit at a Dental Practice

**DOI:** 10.3390/diagnostics15070911

**Published:** 2025-04-02

**Authors:** Lydia Vazquez, Anna Muresan, Cristina Zarauz

**Affiliations:** 1 Department of Orofacial Rehabilitation, University Clinics of Dental Medicine (CUMD), University of Geneva, 1211 Geneva, Switzerland; cristina.zarauz@unige.ch; 2 Barras & Associates, Avenue de France 6, 1950 Sion, Switzerland

**Keywords:** dental radiography, dental radiographs, intraoral X-rays, digital radiology, image quality, radiation protection, radiation shielding, rectangular collimator, rectangular collimation, thyroid gland

## Abstract

**Background/Objectives**: Rectangular collimation (RC) reduces patient radiation exposure but is uncommonly used due to cone-cut concerns. An audit at a dental practice was conducted to analyze impact of RC on the quality of intraoral radiographs. **Methods**: Four X-ray tubes with RC were used. 360 intraoral X-rays were collected, blinded and scored to pre-set qualitative criteria: maximum 14 points for bitewing radiographs (BWs), and 13 for periapical radiographs (PAs). Quality of the X-rays was assessed. **Results**: We found that 48.1% were acceptable, 32.5% were unacceptable and only 19.4% were good X-rays. The loss of image quality was unrelated to RC. Three cone-cuts occurred in PAs without RC. The mean scores for PAs performed without or with RC were as follows: 9.3 ± 1.9 points and 9.6 ± 1.9 points, respectively (*p* = 0.166). The mean scores for BWs performed without or with RC were as follows: 8.0 ± 1.9 points and 7.1 ± 1.9 points, respectively (*p* < 0.001). All scores declined over time. **Conclusions**: This audit highlighted the need for refresher training on film-holder use and the importance of regular maintenance of dental X-ray equipment. Decline in X-ray quality over time was related to wear and tear of X-ray equipment, incorrect image contrast, and technical errors unrelated to RC. No cone-cuts occurred when using RC.

## 1. Introduction

Dental X-rays are a fundamental tool in dentistry. More than one billion dental radiographs are performed each year around the world [1]. The effective dose of one intraoral X-ray is low (0.3 to 22 microsieverts), but low-dose exposure cumulatively can lead to cytotoxic and genetic alterations in sensitive tissues and organs such as the thyroid and the salivary glands [2,3,4]. In total, 300 million intraoral X-rays were taken without a rectangular collimator in dental practices in the United States in 2019, potentially leading to more than 800 radiation-induced cancers [5].

Dental X-rays expose the thyroid gland to the primary X-ray beam and to internal scatter radiation [6]. Abdominal shielding is no longer required for dental X-rays but protection of the thyroid gland if exposed to primary and scatter radiation may benefit the patient. The Swiss Dentomaxillofacial Radiology Society in 2023 recommends thyroid shielding for cephalometric and intraoral radiographs only for patients who have not finished growing [7], whereas the American Dental Association in 2024 advocates that lead aprons and “thyroid shielding during diagnostic intraoral, panoramic, cephalometric, and CBCT imaging no longer should be used in routine practice for pediatric or adult patients” [6] (p. 289). The International Atomic Energy Agency states that “thyroid collars should be used in all examinations where the thyroid may be exposed to the main beam or to a considerable amount of scatter radiation” [4].

Rectangular collimation, proper selection of exposure parameters and correct X-ray imaging technique reduce the patient’s dose at least as much as a thyroid shield [8]. Rectangular collimation, a simple technical adjustment, has been strongly recommended for routine intraoral X-rays because the surrounding tissues, including the thyroid and salivary glands, are less exposed than when round collimation is used [4,6,9,10,11]. Furthermore, rectangular collimation without a thyroid collar minimizes thyroid exposure more effectively than round collimation with a thyroid collar [10]. The use of rectangular collimation when taking intraoral X-rays reduces the patient’s radiation dose by 40 to 92% compared to round collimation [3]. For intraoral adult imaging, the X-ray beam at the end of the rectangular collimator should ideally be limited to no more than 35 by 45 mm [8].

Despite the evidence for the effectiveness of rectangular collimation, its widespread adoption has been hindered by concerns about cone-cuts in favor of the more commonly used round collimation. When rectangular collimation is used by dental students, low cone-cut rates and low re-exposure rates have recently been reported, thus encouraging the use of this beam reduction device for student training [12,13]. Moreover, studies suggest that the dose reduction justifies the use of rectangular collimation even if cone-cuts may result [14].

Good-quality X-rays are fundamental for accurate diagnosis. X-ray quality can be improved with contributions from clinical audits. Whereas clinical research investigates clear questions and hypotheses in a systematic way to “generate new evidence to refute, support or develop a hypothesis” [15] (p. 3), the purpose of a clinical audit in healthcare is to improve clinical practice. Clinical audits are cyclical. First, peer dentists and doctors examine current practices and make recommendations for improvement. The implemented changes are later followed-up and re-evaluated with a second audit followed by feedback and recommendations for improvement as necessary. Patient-related audits in dentistry include an audit of patient satisfaction by survey, audit of dental caries management in children, and audit of the quality of BWs and periapical X-rays [16]. The latter was selected for a dental practice. The primary goal for using rectangular collimation is to reduce the patient radiation dose but that can be compromised if the X-ray has no value for diagnostic purposes.

The purpose of this audit at a private dental practice was to evaluate the impact of rectangular collimation on the quality of intraoral X-rays, to identify deficiencies and factors affecting image quality, and to recommend corrective action to improve patient care. The null hypothesis was that the use of rectangular collimation would not significantly affect the quality of intraoral X-rays.

## 2. Materials and Methods

### 2.1. Materials

The audit retrospectively assessed the image quality of intraoral X-rays to determine adherence to best practices. Specifically, it evaluated whether dental X-ray image quality was maintained following the introduction of rectangular collimators and whether the number of cone-cuts increased.

The clinical audit was conducted at a Swiss dental practice selected for the variety of rectangular collimators fitted to four X-ray tubes and for the diversity of oral healthcare providers taking intraoral X-rays.

The sample consisted of bitewing radiographs (BWs) and periapical radiographs taken by dentists, dental hygienists, dental assistants and dental prophylaxis assistants. All oral healthcare providers were trained in the use of film-holders and the parallel technique required when using a rectangular collimator. Only X-rays taken with sizes 1 and 2 digital imaging plates on adults were collected during a pre-set time period.

**Figure 1:**
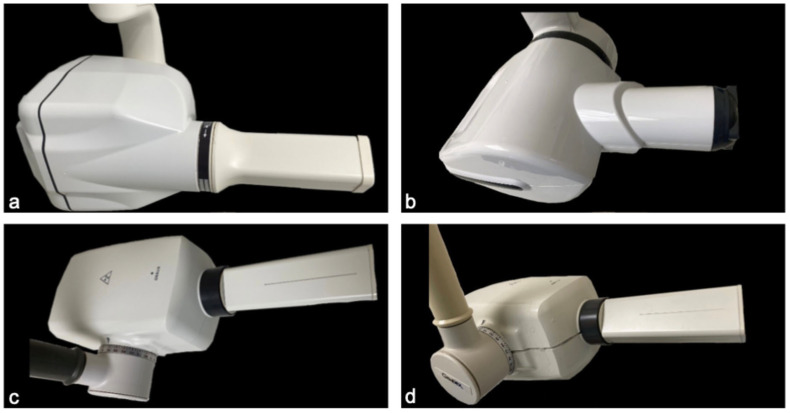
The four X-ray tubes with their respective rectangular collimators in place. From left to right and top to bottom: (**a**) Xmind AC (Acteon Group, Mérignac, France); (**b**) CS2200 (Carestream Dental, Atlanta, GA, USA); and (**c**,**d**) Oralix AC (Gendex/KaVo Dental, Biberach, Germany).

The X-ray imaging system consisted of one Digora Optime photostimulable phosphor scanner (Soredex, Helsinki, Finland) and Digora photostimulable storage phosphor plates (hereafter named digital plates). Four X-ray generators (Figure 1) were used. Each generator was equipped with a rectangular collimator of the same brand as the generator. All X-ray images were taken using Rinn XCP film-holders and centering rings provided by Dentsply (Sirona, Bensheim, Germany) to standardize intraoral X-ray techniques. These film-holders feature a centering ring with four notches to ensure optimal alignment of the rectangular collimator, i.e., the X-ray cone perpendicular to the digital plate.

The audit was part of a Master’s thesis by a student in dental medicine (mentored by an expert in dentomaxillofacial radiology). The protocol was submitted to Swissethics, the Swiss association of research ethics committees, who determined that the present audit did not require ethical approval (Req-2022-01478).

### 2.2. Methods

#### 2.2.1. Image Collection

A total of 360 anonymized intraoral radiographs were collected (180 BW and 180 periapical radiographs).

The X-ray images were exported in JPEG format, sorted by acquisition date, and divided into 8 groups based on collimator type (rectangular or round) and on the time relative to collimator introduction (Table 1):

- Groups A and E: Before collimator introduction;
- Groups B–D (PA) and F–H (BW): 0–6 months (B and F), 6–12 months (C and G), and >12 months (D and H) post collimator introduction, for PA and BW radiographs, respectively.

#### 2.2.2. Observer Calibration and Blinding

One observer (A.M.) was calibrated to assess image quality by scoring X-rays extracted from a database of anonymized intraoral radiographs. The calibration process involved scoring radiographs using predefined quality criteria (Table 2 and Table 3) and discussing discrepancies with the supervising radiology expert (L.V.).

For blinding, an independent observer (not trained in the dental field and uninvolved in the audit) compiled the radiographs into a PowerPoint presentation, assigning each image a randomly generated unique identifier. All group information (Table 1) was removed, ensuring the primary observer (A.M.) was blinded to collimation type and acquisition date during scoring.

#### 2.2.3. Image Quality Assessment

X-rays were evaluated once (A.M) according to pre-set qualitative criteria for periapical (Table 2) and BW (Table 3) radiographs. These tables were modeled on tables of quality criteria reported in a recent study at a Swiss dental school [12].

**Figure 2:**
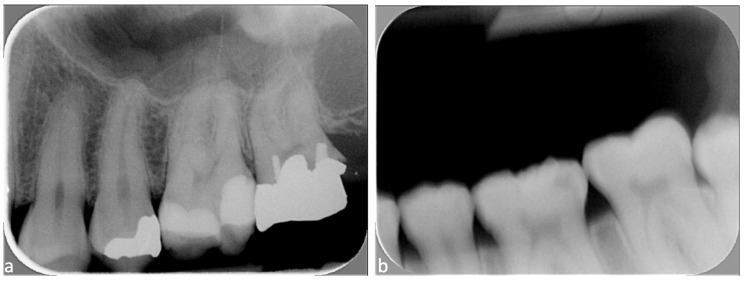
Periapical radiograph scores presented from left to right: (**a**) score 11/13: acceptable X-ray; and (**b**) score 3/13: unacceptable X-ray.

Following evaluation, the independent observer reassigned acquisition dates and collimation status to each image. All scores were entered into an Excel database to calculate mean scores and trends over time for each group (A to H). Maximum score was 13 points for periapical radiographs; periapical radiographs with a score equal to or greater than 12 points were considered good X-rays, those with a score between 8 and 11 points were acceptable X-rays, and those with a score less than or equal to 7 qualified as unacceptable X-rays (Figure 2).

**Figure 3:**
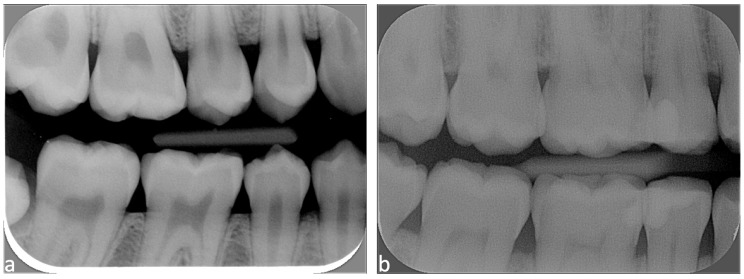
Bitewing scores presented from left to right: (**a**) score 11/14: good X-ray; and (**b**) score 4/14: unacceptable X-ray.

The maximum score was 14 points for BWs; BWs with a score equal to or greater than 11 points were considered good X-rays, those with a score between 8 and 10 were acceptable, and BWs with a score less than or equal to 7 were considered unacceptable X-rays (Figure 3).

### 2.3. Statistical Analysis

Given the audit’s exploratory nature and sample size (90 cases for each control group and 30 for each test group), a priori power analysis was not conducted. The Shapiro–Wilk test was applied to evaluate the normality of the sample. Non-normal distributions were observed; hence, Kruskal–Wallis tests were conducted. Post hoc pairwise comparisons were conducted using Dunn’s test with Bonferroni correction for multiple comparisons. Levene’s test to assess homogeneity of variance was conducted. A 5% significance level (α = 0.05) was applied to all analyses. Statistical analyses were conducted using SPSS software (v30; IBM Corp., Chicago, IL, USA).

## 3. Results

### 3.1. Overall Analysis

The results from Levene’s test showed *p*-values > 0.05 for both PA and BW radiographs, confirming that the assumption of homogeneity of variance was met.

When analyzing the total scores for the periapical radiographs, results showed no significant difference between control (A) and test groups (B, C and D) (*p* = 0.166). However, for BWs, a significant difference was observed (*p* < 0.001) leading to a partial rejection of the null hypothesis.

**Figure 4:**
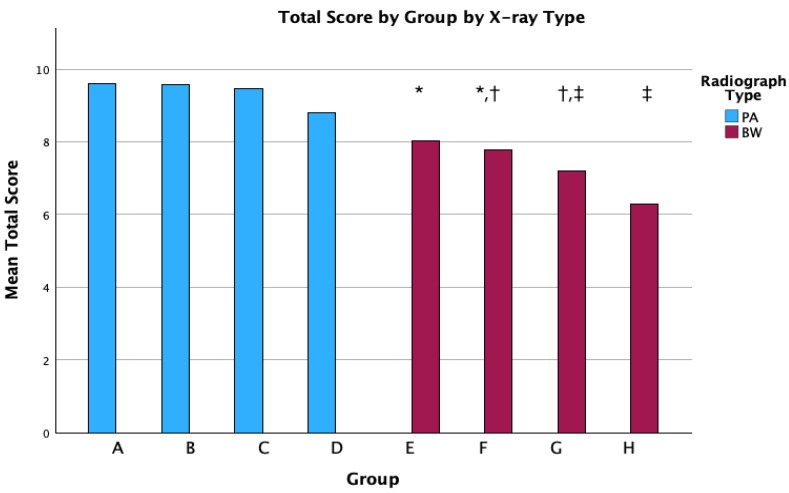
Bar graph representing the mean total scores achieved in each group (A–H). Blue bars represent periapical radiographs (PAs), while red bars represent bitewing radiographs (BWs). Significant differences were shown between BW groups and are represented with symbols (*, †, and ‡). Different symbols mean significant differences between groups with *p*-value *p* < 0.05.

A post hoc analysis revealed significant differences between groups E–G, E–H, and F–H. The evolution over time of the mean total scores for each group and the significant differences are shown in a bar graph (Figure 4).

The number and percentage of good, acceptable, and unacceptable X-rays are detailed for each group in Table 4.

The descriptive data, detailing the number and percentage with a complete score for each evaluated criterion across all groups, are presented in Table 5 for periapical radiographs and in Table 6 for BWs. The mean and standard deviation (SD) of the total scores for each group are also included in these tables. Individual quality parameters were evaluated. Among the positioning-related variables, quadrant ratio and vertical distortion demonstrated significant differences between groups that are shown in Table 5 (periapical radiographs) and Table 6 (BWs). When evaluating the image quality, all three categories—image contrast (*p* = 0.009), foreign object ambient light (*p* < 0.001), and scratches (*p* < 0.001)—showed significant differences among groups. Differences between groups are shown in Table 5 (periapical radiographs) and Table 6 (BWs).

### 3.2. Quality Analysis of Periapical Radiographs

**Figure 5:**
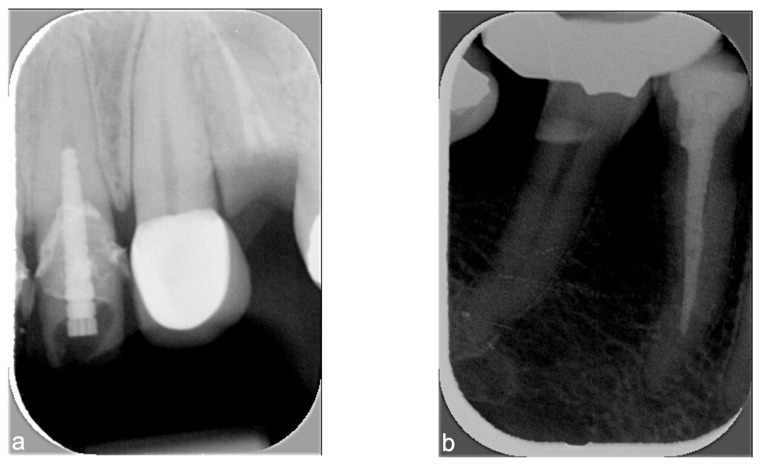
Periapical radiographs taken without rectangular collimation. From left to right: (**a**) incorrect bite on the film-holder (apex has been “cut off”) and (**b**) an overexposure error.

The number of truncated periapical radiographs was 3 out of 360 (0.8%) of the total sample, corresponding to 3 out of 90 (3.3%) of the periapical radiographs taken without rectangular collimation. Incorrect bite on the film-holder, incomplete visibility of the tooth (tooth not fully visible from crown to apex), followed by image contrast error were the most frequent parameters with incomplete scores on periapical radiographs taken without rectangular collimation (Figure 5).

**Figure 6:**
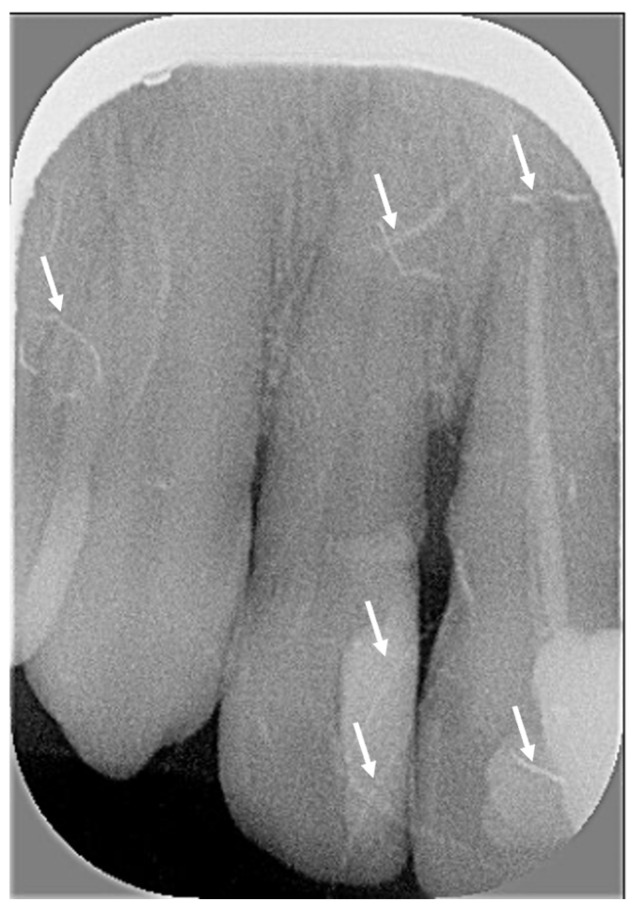
Grainy low-contrast X-ray with multiple radiopaque scratch artefacts (shown with white arrows).

When analyzing the ambient light exposure and image contrast criteria for periapical radiographs, the observer noted that several X-rays were “problematic images” i.e., image quality was severely affected. “Problematic images” were very blurred or pixelated, lacking image contrast (Figure 6). They were scored by audit as lacking contrast.

**Figure 7:**
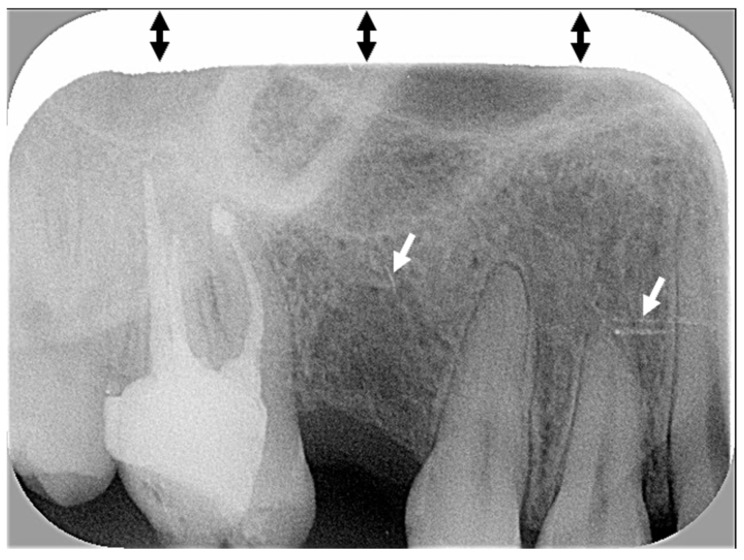
A truncated X-ray due to poor calibration of the digital plate scanner (black double arrows). Scratches are shown with white arrows.

**Figure 8:**
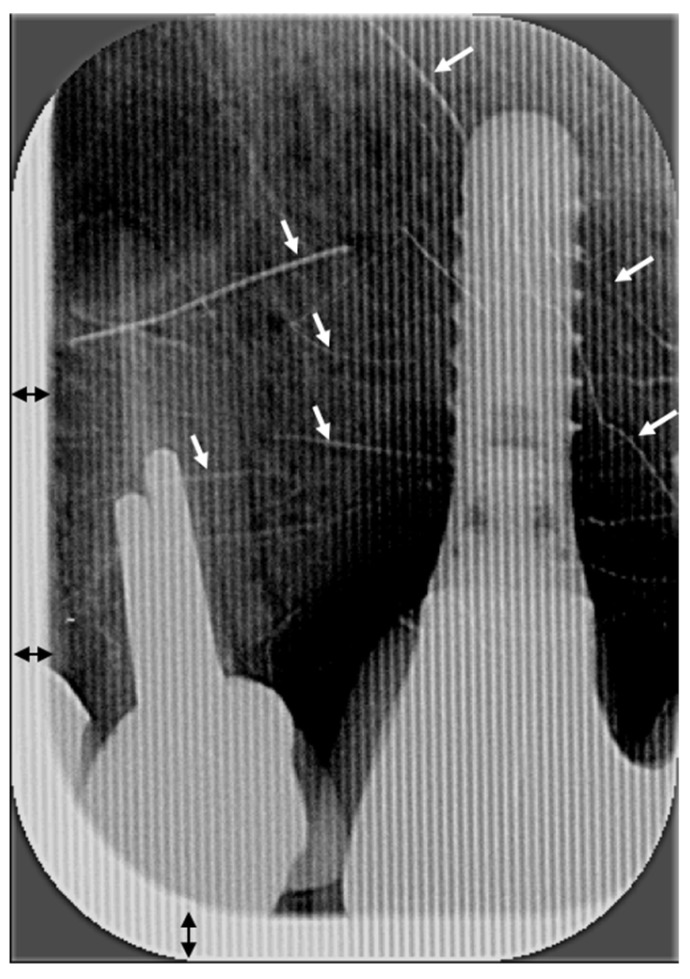
Vertical dark grey and white stripes superimposed on the dental implant, neighboring tooth, and dentoalveolar structures are due to malfunction of the digital plate scanner resulting in important loss of image quality. Multiple thin white scratches (shown with white arrows) in all directions are also visible. The truncated part of the X-ray, due to poor calibration of the photostimulable phosphor scanner, is shown with double black arrows.

There was a high number of truncated images (Figure 7) related not to incorrect positioning of the rectangular collimator but to poor calibration of the Digora Optime photostimulable phosphor scanner. Vertically striped images (Figure 8), due to malfunction of the digital plate scanner, resulted in important loss of image quality. Vertically striped images were scored by audit as presenting a foreign object.

### 3.3. Quality Analysis of BW

**Figure 9:**
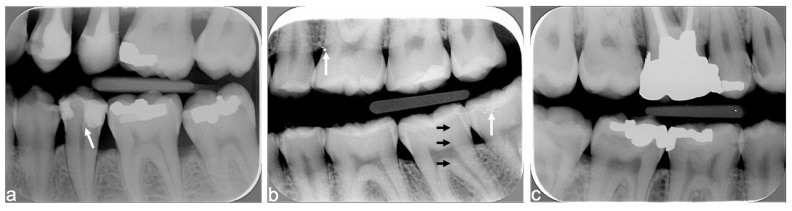
Bitewing (BW) X-rays taken with rectangular collimation. BW on the left (**a**) shows an incorrect quadrant ratio (the maxillary quadrant is less visible than the mandibular quadrant). The BW on the right (**c**) shows a correct quadrant ratio (almost identical visibility of teeth and alveolar crest at vertical level). Figure (**a**) shows correct visibility of the distal contact points of the lower canine and 2nd molars, whereas BWs (**b**,**c**) show a “distal contact points” error (i.e., lower canine and first lower premolar are missing on the BWs). The middle BW (**b**) shows several artefacts: a white line across the top (due to poor calibration of the digital plate scanner), several radiopaque speckled scratches and, in addition, a discrete vertical dark grey line (shown with black arrows, not to be diagnosed as a tooth fissure) appears to cut the second lower molar in two. Scratches (shown with white arrows) are visible.

No truncation errors were observed on BWs taken with or without rectangular collimation. Examples of three BWs taken with rectangular collimation that achieved less than the maximum total score are shown in Figure 9.

## 4. Discussion

Empowering oral healthcare providers to recognize and correct errors on dental X-rays improves clinical practice: good-quality dental X-rays lead to better diagnostic performance and better patient care. The purpose of a clinical audit in healthcare is to improve practice through peer-review of current clinical practice [15]. When a new tool is introduced at a dental practice, its impact on patient care should be examined. The audit examined the factors affecting the quality of intraoral X-rays following the introduction of rectangular collimation. Out of 360 X-rays scored, 48.1% were deemed acceptable, 32.5% were unacceptable and only 19.4% were scored as good X-rays. There were 4 times as many unacceptable BWs (95/180, 52.8%) than unacceptable periapical radiographs (22/180, 12.2%). The percentages of good and acceptable periapical radiographs taken with rectangular collimation (27.8% and 58.9%, respectively) were similar to those taken without (32.2% and 56.7%, respectively). The percentages of good and acceptable BWs taken with rectangular collimation (6.7% and 30.0%, respectively) were much lower than those taken without (11.1% and 46.7%, respectively).

The number of periapical radiographs that achieved the maximum total score declined over time. Periapical radiographs taken without rectangular collimation (Group A control group) obtained a slightly better mean score (9.6 ± 1.9 points) than those taken with rectangular collimation (mean score of 9.3 ± 1.9 points). The mean score for periapical radiographs taken with rectangular collimation was 9.6 ± 1.9 points through the first six months (Group B), 9.5 ± 2.1 points between 6 to 12 months (Group C), and 8.8 ± 1.9 points more than a year after introduction of rectangular collimation (Group D). Thus, image quality for periapical radiographs taken with rectangular collimators initially remained similar to that before their introduction, likely due to the staff’s focused attention when using a new tool to obtain X-rays. However, as handling became routine, image quality deteriorated. The same trend was observed for BWs. BWs taken without rectangular collimation (Group E control group) achieved a higher mean score (8.0 ± 1.9 points) than BWs taken with rectangular collimation (7.1 ± 1.9 points). For BWs taken with rectangular collimation, the mean score was 7.8 ± 2.1 points through the first six months (Group F), then decreased slightly (7.2 ± 1.9 points) between 6 to 12 months (Group G), and then decreased drastically to a score of 6.3 ± 1.7 points (indicating unacceptable X-rays) more than a year after introduction of rectangular collimation (Group H).

Thus, following introduction of rectangular collimation, overall quality of intraoral X-rays deteriorated, suggesting that current practice when taking intraoral X-rays, especially BWs, ought to be revised to improve image quality. Several factors that may explain the loss of image quality over time were examined: difficulties centering the rectangular collimator in the horizontal and vertical planes, centering errors of the digital plates, other handling mistakes (such as exposure parameter errors) and wear and tear of X-ray equipment.

For periapical radiographs taken with rectangular collimation, the loss of image quality was mainly attributed to incorrect image contrast (detected in 45.6% of periapical radiographs taken with rectangular collimation), incorrect bite on the film-holder (41.1%), incomplete visibility of the tooth (41.1%), and digital plate artefacts (i.e., scratched, cracked or detached edges on digital plates visible in 40% of periapical radiographs taken with rectangular collimation). Digital plate artefacts were detected in only 15.6% of periapical radiographs taken prior to rectangular collimation introduction. Low image contrast was due to questionable management of exposure parameters or degradation of image resolution caused by aging digital plates and aging photostimulable phosphor scanner.

For BW radiographs taken with rectangular collimation, the decline in scores over time was observed in the digital plate quality parameters and other parameters unrelated to cone centering parameters leading to cone-cuts. For BWs, the loss of image quality was most often related to poor centering of the digital plate in the film-holder (resulting in unequal visibility of one quadrant compared to the other in 92.2% of BWs taken with rectangular collimation), absence of visible proximal contact points (distal to the lower canine not visible in 91.1% of BWs taken with rectangular collimation), overlap of contact points related to horizontal angulation error (wrong angulation of either the film-holder or the X-ray tube, or both detected in 83.3% of BWs), incorrect image contrast (detected in 55.6% of BWs taken with rectangular collimation) and incorrect bite on the film-holder (in 42.2% of BW taken with rectangular collimation). This suggests quality of BWs was affected by parameters unrelated to rectangular collimation. Various studies have reported common errors on X-rays. Projection geometry errors can account for up to 31.9% of X-rays [17]. X-rays taken by dental students showed improper vertical angulation or film placement (12.5% and 49.9%, respectively) [18] and another study reported 22.4% projection geometry errors and 30.4% incorrect film placement by dental students and dental staff [19]. Projection geometry errors were found/observed in 21% of periapical radiographs and 77% of BWs scored in this audit.

The number of cone-related truncated X-rays was very low (3/360, 0.8% of X-rays) in this audit. The cone-cuts detected on 3 periapical radiographs were taken without rectangular collimation. Cone-cuts have been reported in 20.8% of X-rays taken by dental students [18], and 39.5% and 21% of X-rays taken by dental students and staff [13,19]. Under the conditions of this audit, the much-apprehended truncation caused by rectangular collimation (potentially resulting in an unacceptable image quality for diagnostic purposes and requiring re-exposure) seemed not justified. The use of film-holders with centering rings at the dental practice likely helped prevent cone-cuts on X-rays, despite the rectangular collimation in use. The results from this audit suggest that the staff targeted the digital plates more attentively when the rectangular collimators were first introduced. No images were truncated by the rectangular collimators and their use does not explain the decline in image quality over time.

Despite a significant increase in truncated X-rays with rectangular collimation, re-exposure rates did not increase significantly because errors did not produce unacceptable images [13,20]. Previous studies reported that 2.7% [18] to 3% [13] of radiographs were not usable due to cone-cuts, which led to retakes. The present audit sought to analyze image quality only, not the re-exposure rate. Nonetheless, some low-scoring X-rays (e.g., Figure 5 and Figure 8) likely required retakes. In the Swiss dental school study, only one radiograph, taken without rectangular collimation, needed to be repeated, representing a very low re-exposure rate (1/130, 0.77%) [12]. In that study, 20% (26/130) of X-rays were truncated, with nearly 50% (14/26) showing a white horizontal line in the upper or lower margin due to poor calibration of one of the photostimulable phosphor scanners; radiographs of a step wedge confirmed scanner malfunction [12].

Photostimulable phosphor scanner failure can cause various errors, including image fragmentation, sandy views, and edge-masking defects, which may be corrected by upgrading the software or recalibrating the scanner [17]. Poor calibration of the photostimulable phosphor scanner leading to truncated images was also observed in the present audit. Indeed, when a photostimulable phosphor scanner is not properly calibrated, X-ray images are horizontally or vertically cut off by a few millimeters, regardless of staff handling the digital plate, centering of the X-ray tube or exposure of the digital plate. The photostimulable phosphor scanner at the dental practice required recalibration to correct scanner-related image cuts.

“Problematic images” such as blurred or pixelated X-rays (low image resolution), low contrast images, vertical dark grey lines and a high number of horizontally truncated images (unrelated to the incorrect positioning of the rectangular collimator) were observed in X-rays performed several months after introduction of rectangular collimation. The poor quality of those X-rays was due to degraded Digora Optime photostimulable phosphor scanner and digital plates. The percentage of digital plate artefacts (i.e., X-rays with traces of scratches, folds or other marks with various widths and lengths) observed on periapical radiographs increased considerably over the four time periods: 15.6% (group A), 20% (group B), 50% (group C) and 50% (group D). An in vitro study showed that, after just 50 uses, scratches in digital imaging plates can compromise the quality of X-rays [21]. The authors of that study also noted that scratches can require retakes, unnecessarily exposing the patients to radiation or compromising treatment if diagnostic information is obscured [21]. Other studies have shown that pixel grey values of X-rays decreased after several exposures and scans of the digital imaging plates [21,22,23]. In this audit, “problematic images” were more frequently reported in periapical groups C and D (6 and 12 months after introduction of rectangular collimation, respectively) than in earlier groups (A and B). For BWs, the percentage of digital plate artefacts increased dramatically after the introduction of rectangular collimation: 6.7% (Group F), 16.7% (Group G), and 56.7% (Group H). These findings are unsurprising because the same size 2 digital plates were used for both BW and posterior periapical radiographs. Digital plates have a limited lifespan. Digital plates have a limited lifespan and manufacturers recommend replacement after a few hundred exposures. Some authors suggest that digital plates can be used up to 1000 times before their grayscale degrades [23]. Although the exact number of exposures per digital plate was not tracked in this audit, the loss of image contrast, along with the increasing number of scratches and artefacts over time, highlights the importance of maintaining equipment. Importantly, problematic images were not caused by rectangular collimation but by the wear and tear of aging equipment, which negatively affected image quality. A simple way to verify the image quality is with an X-ray of a step wedge. “Subsequent radiographs of the step wedge should be made during clinical use and compared with the reference one to ensure that image quality is maintained” [4].

The audit revealed the number of good, acceptable, and unacceptable X-rays. Importantly, various factors led to unacceptable image quality. Feedback and actionable steps to avoid loss of image quality shall be given to the dental team. First, their Digora Optime photostimulable phosphor scanner should be thoroughly evaluated, and all digital plates should be tested with a step wedge (or equivalent) and probably replaced with new ones. The four X-ray tubes should also be evaluated. Regular quality control followed by proper maintenance of X-ray equipment is essential [4]. Second, attention should be given to proper exposure parameters and to the timely replacement of deteriorating digital plates. Image artefacts, particularly multiple radiopaque scratches on X-rays, should be monitored because they indicate that digital plates may need replacing. For BWs, correct centering of the digital plate in the film-holder and ensuring proper bite alignment are essential for proper quadrant visibility. To properly visualize the full surface of all four premolars and first and second molars on BWs, the staff should learn to position the film-holder in the oral cavity and to project the distal contact point of the lower canine on the digital plate. Excellence is achieved with training and monitoring of image quality.

The limitations of this audit include the analysis of only a small sample of X-rays by a single observer due to time and resource constraints. The audit primarily focused on factors leading to a loss of X-ray quality related to the introduction of rectangular collimation. A follow-up re-audit, which may also evaluate the re-exposure rate, is planned to assess any improvements made at the dental practice since the first audit. Due to limitations in controlling for confounding variables (e.g., radiographic settings), future audits should investigate a larger sample size under more controlled conditions using a multivariate regression analysis. The X-ray scoring system indicated the changes necessary to improve image quality but the dental school’s scoring system may require adapting for circumstances in private dental practice. The dental school’s scoring system may be too specific for a private dental practice where a wide range of staff perform X-rays generating diagnostically usable X-rays without achieving the gold standard expected of university dental students. A simpler grading system may be more appropriate for a re-audit to accommodate a broader range of staff expertise. Furthermore, a subanalysis of the impact of each rectangular collimator was not feasible because anonymization of the X-rays precluded identifying which collimator model was used for each X-ray. Anonymization also precluded the assessment of dental staff experience on image quality; more experienced dentists may handle exposure parameters better than dental assistants. Future audits might explore factors not examined in this audit such as digital plate handling and staff training and experience.

## 5. Conclusions

The main findings of the audit indicate that dental X-ray quality was affected by various factors such as wear and tear of digital plates, malfunction of the photostimulable phosphor scanner, incorrect image contrast and other technical errors, which were unrelated to rectangular collimation. Overall, the quality of both BWs and periapical X-rays declined over time. No cone-cuts were observed on BW or periapical radiographs taken with rectangular collimators at the private dental practice. This audit emphasizes the need for refresher training on film-holder use for dental staff and the importance of regular maintenance of dental X-ray equipment. A re-audit, including a larger sample of X-rays and a simplified X-ray scoring system, is recommended.

## Figures and Tables

**Table 1:** Distribution of 360 bitewings and periapical radiographs divided into 8 groups (A to H). Groups A and E include X-rays taken without rectangular collimator. Groups B–D and F–H represent different time periods following introduction of rectangular collimation.

Group	X-Ray Type and Period They Were Taken	Number
A	PAs without rectangular collimator (control group)	90
B	PAs < 6 months after rectangular collimator introduction	30
C	PAs 6–12 months after rectangular collimator introduction	30
D	PAs > 1 year after rectangular collimator introduction	30
E	BWs without rectangular collimator	90
F	BWs < 6 months after rectangular collimator introduction	30
G	BWs 6–12 months after rectangular collimator introduction	30
H	BWs > 1 year after rectangular collimator introduction	30
	Total	360

BWs = bitewing radiographs; PAs = periapical radiographs.

**Table 2:** Scores for the quality analysis of periapical radiographs (modeled on [12]).

Parameter	Criteria	2-Points Score	1-Point Score	0-Point Score
Cone centering (with or without rectangular collimator)	Image outline Tooth fully visible	Complete image outline; borders not cut No truncated image	Incomplete image outline; image cut without consequences on diagnosis	Incomplete image outline; image cut with consequences on diagnosis
Centering of the digital plate	Target tooth: >2 mm of periapical bone is visible AND visible periapex of adjacent anterior tooth (size 1) or two adjacent teeth (size 2)	--	Yes	No (one of the two parameters is not fulfilled)
	Mesial and distal view of front tooth (size 1), or of two adjacent teeth (size 2)	--	Yes	No
	Tooth fully visible (from crown to apex)	--	Yes	No
	Correct bite on the film-holder	--	Yes	No
Axis of the digital plate	Correct axis of plate	--	Yes	No
No vertical distortion	No distortion (absence of tooth shortening or lengthening)	--	Yes	No
Image contrast	Correct image contrast	--	Yes	No (too bright or too dark)
Artefacts	Exposure to ambient light, presence of foreign object or vertical stripes	--	No Or minimal without consequence on diagnosis	Yes With consequence on diagnosis
	Intact digital plate, without traces of scratches, folds, or other	--	No Or minimal without consequence on diagnosis	Yes With consequence on diagnosis
Overall X-ray quality	Overall quality	Excellent overall quality	Acceptable quality; minor errors	Major error; interpretation is difficult

**Table 3:** Scores for the quality analysis of bitewing radiographs (modeled on [12]).

Parameter	Criteria	2-Points Score	1-Point Score	0-Point Score
Centering the cone with or without rectangular collimator	Image outline Tooth fully visible	Complete image outline; borders not cut No truncated image	Incomplete image outline; image cut without consequences on diagnosis	Incomplete image outline, image cut, with consequence on diagnosis
Centering of the digital plate	Ratio of maxillary and mandibular quadrants (visibility of teeth and alveolar crest at vertical level)	50–50% ratio: image well centered vertically (maxillary and mandibular teeth each cover 50% of the image)	40–60% ratio: image moderately centered vertically (one quadrant covers more than half of the image, alveolar bone visible on both quadrants)	Image not vertically centered (one quadrant covers more than 60% of the image, alveolar bone of opposite quadrant not visible)
	Distal contact points (between canines and the 2nd lower molars)	4 distal contact points are visible	3 distal contact points are visible	<3 distal contact points are visible
	Correct bite on the film-holder	--	Yes Upper and lower quadrants in contact with film-holder	No One quadrant without contact with film-holder
Superposition of enamel (horizontal angulation)	Superposition of enamel on the contact points	Superposition <1/3 of enamel width	Superposition between 1/3 and 2/3 of enamel width	Superposition >2/3 of enamel width
Image contrast	Correct image contrast	--	Yes	No (too bright or too dark)
Artefacts	Exposure to ambient light, presence of foreign object or vertical stripes	--	No Or minimal without consequence on diagnosis	Yes With consequence on diagnosis
	Intact digital plate, without traces of scratches, folds, or other	--	No Or minimal without consequence on diagnosis	Yes With consequence on diagnosis
Overall X-ray quality	Overall quality	Excellent overall quality	Acceptable quality; minor faults/errors	Major error; diagnosis made difficult

**Table 4:** Number and percentage of good, acceptable, and unacceptable periapical radiographs and bitewings taken with or without a rectangular collimator. Maximum score was 13 points for periapical radiographs (≥12 good; 8–11 acceptable; and ≤7 unacceptable). Maximum score was 14 points for BW (≥11 good; 8–10 acceptable; and ≤7 unacceptable).

Group	X-Ray Type and Period They Were Taken	Good	Acceptable	Unacceptable
A	Total PAs without RC	29/90 (32.2%)	51/90 (56.7%)	10/90 (11.1%)
B	PAs < 6 months after RC introduction	8/30 (26.7%)	21/30 (70.0%)	1/30 (3.3%)
C	PAs 6–12 months after RC introduction	11/30 (36.7%)	13/30 (43.3%)	6/30 (20.0%)
D	PAs > 1 year after RC introduction	6/30 (20.0%)	19/30 (63.3%)	5/30 (16.7%)
	Total PAs with RC	25/90 (27.8%)	53/90 (58.9%)	12/90 (13.3%)
				
E	Total BWs without RC	10/90 (11.1%)	42/90 (46.7%)	38/90 (42.2%)
F	BWs < 6 months after RC introduction	3/30 (10.0%)	12/30 (40.0%)	15/30 (50.0%)
G	BWs 6–12 months after RC introduction	3/30 (10.0%)	8/30 (26.7%)	19/30 (63.3%)
H	BWs > 1 year after RC introduction	0/30 (0.0%)	7/30 (23.3%)	23/30 (76.7%)
	Total BWs with RC	6/90 (6.7%)	27/90 (30.0%)	57/90 (63.3%)
				
	Total PAs	54/180 (30.0%)	104/180 (57.8%)	22/180 (12.2%)
	Total BWs	16/180 (8.9%)	69/180 (38.3%)	95/180 (52.8%)
	Total X-rays	70/360 (19.4%)	173/360 (48.1%)	117/360 (32.5%)

BWs = bitewing radiographs; PAs = periapical radiographs; RC = rectangular collimator.

**Table 5:** Number and percentage of parameters with a complete score for periapical radiographs.

PA Groups	Number of X-Rays n	Cone Centering n (%)	Visible Periapex n (%)	M and D Views n (%)	Tooth Fully Visible n (%)	Bite on Film-Holder n (%)	Axis plate n (%)	No Vertical Distortion n (%)	Image Contrast n (%)	Foreign Object Ambient Light n (%)	Plate Artefacts/Scratches n (%)	Mean (SD)	Total Score in % Mean (SD)
A	90	87 (96.7)	74 (82.2)	80 (88.9)	50 (55.6)	43 (47.8)	74 (82.2)	68 * (75.6)	51 (56.7)	81 * (90)	76 * (84.4)	9.6 (1.9)	73.9 (14.6)
B	30	30 (100)	26 (86.7)	28 (93.3)	17 (56.7)	14 (46.7)	24 (80)	25 * (83.3)	19 (63.3)	21 † (70)	24 * (80)	9.6 (1.6)	73.9 (12.3)
C	30	30 (100)	27 (90)	29 (96.9)	18 (60)	20 (66.7)	29 (96.9)	26 * (86.7)	18 (60)	13 ‡ (43.3)	15 † (50)	9.5 (2.1)	73.1 (16.2)
D	30	30 (100)	25 (83.3)	28 (93.3)	18 (60)	19 (63.3)	24 (80)	13 † (43.3)	12 (40)	26 *,† (86.7)	15 † (50)	8.8 (1.9)	67.7 (14.6)
Total B to D	90	90 (100)	78 (86.7)	85 (94.4)	53 (58.9)	53 (58.9)	77 (85.6)	64 (71.1)	49 (54.4)	60 (66.7)	54 (60)	9.3 (1.9)	71.5 (14.6)

PA = periapical radiograph; n = number; parameters showing significant differences between groups are displayed with symbols (*, †, and ‡). Different symbols represent significant differences between groups with *p*-value *p* < 0.05.

**Table 6:** Number and percentage of parameters with a complete score for bitewing radiographs.

Bitewing Groups	Number of X-Rays n	Cone Centering n (%)	Quadrant Ratio n (%)	Distal Contact Points n (%)	Bite on Film-Holder n (%)	Horizontal Angulation n (%)	Image Contrast n (%)	Foreign Object Ambient Light n (%)	Plate Artefacts/Scratches n (%)	Total Points Mean (SD)	Total Score in % Mean (SD)
E	90	90 (100)	12 * (13.3)	10 (11.1)	36 (40)	26 (86.7)	60 * (66.7)	89 * (98.9)	70 * (77.8)	8.0 (1.9)	57.1 (13.6)
F	30	30 (100)	5 * (16.7)	3 (10)	15 (50)	7 (23.3)	16 * (53.3)	22 † (73.3)	28 * (93.3)	7.8 (2.1)	55.7 (15)
G	30	30 (100)	1 * (3.3)	4 (13.3)	19 (63.3)	3 (10)	16 * (53.3)	11 ‡ (36.7)	25 * (83.3)	7.2 (1.9)	51.4 (13.6)
H	30	30 (100)	1 † (3.3)	1 (3.3)	18 (60)	5 (16.7)	8 † (26.7)	26 ¥ (86.7)	13 † (43.3)	6.3 (1.7)	45 (12.1)
Total F to H	90	90 (100)	7 (7.8)	8 (8.9)	52 (57.8)	15 (16.7)	40 (44.4)	59 (65.6)	66 (73.3)	7.1 (1.9)	50.7 (13.6)

n = number; parameters showing significant differences between groups are displayed with symbols (*, †, ‡, and ¥). Different symbols represent significant differences between groups with *p*-value *p* < 0.05.

## Data Availability

The data can be provided upon request. Please contact the corresponding author.

## Abbreviations

The following abbreviations are used in this manuscript:

BWs	Bitewing radiographs
PAs	Periapical radiographs
RC	Rectangular collimator
